# A Rare Case of a Bladder Neck Abscess Masquerading as a Benign Mass

**DOI:** 10.1155/2022/9966553

**Published:** 2022-10-04

**Authors:** Victor A. Abdullatif, Jacob Novack, Philip J. Shalhoub, Todd G. Campbell, Joel E. Abbott

**Affiliations:** Urology, Ascension Macomb-Oakland Hospital, Warren, 48093 MI, USA

## Abstract

**Introduction:**

Bladder neck abscesses are rare urologic pathologies with very few cases published in modern literature. This report explores a case of a bladder neck mass incidentally found on computed tomography (CT) imaging in a patient with an iliopsoas abscess. *Case Presentation.* We present a case of a 60-year-old woman who was recently treated for sepsis secondary to an iliopsoas abscess in July of 2022. A CT scan revealed an indeterminate structure in the posterior inferior left paramedian bladder wall. During a cystoscopy with transurethral resection of the mass, an abscess was uncovered and evacuated. A postoperative Foley catheter was left in place, and the patient recovered without any complications.

**Conclusion:**

At the time of publication, the patient feels well and denies pain or lower urinary tract symptoms. Although bladder abscesses are exceptionally rare, incidental findings during cystoscopy may warrant further investigation in patients with comorbid abscesses.

## 1. Introduction

As a diagnostic entity, bladder neck abscesses are exceptionally rare anomalies. To date, there is only one report of a bladder wall abscess in the literature [1]. The etiology, epidemiology, clinical features, and management of these entities are not well defined as there are virtually no modern publications on the topic.

A thorough literature review revealed that intramural abscesses of the bladder wall and urachus are only slightly more common than bladder neck abscesses, with urachal abscesses representing a larger majority of these pathologies [2–4]. Due to the paucity of literature reporting their incidence, we describe a case of bladder neck abscess in a female patient. To the best of our knowledge, this is the first case report of bladder neck abscess uncovered incidentally during transurethral resection of a bladder mass. This case report serves to highlight a rare clinical phenomenon in urologic surgery.

## 2. Case Presentation

**Figure 1:**
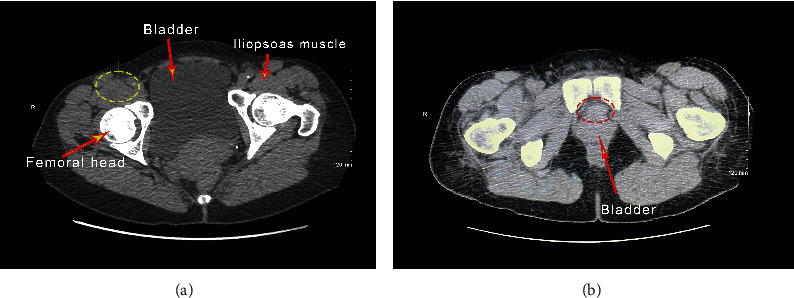
CT scan of the abdomen and pelvis demonstrating a psoas abscess ((a) yellow circle) and a 20 × 15 × 14 mm soft tissue density in the inferior posterior left paramedian bladder wall ((b) red circle).

**Figure 2:**
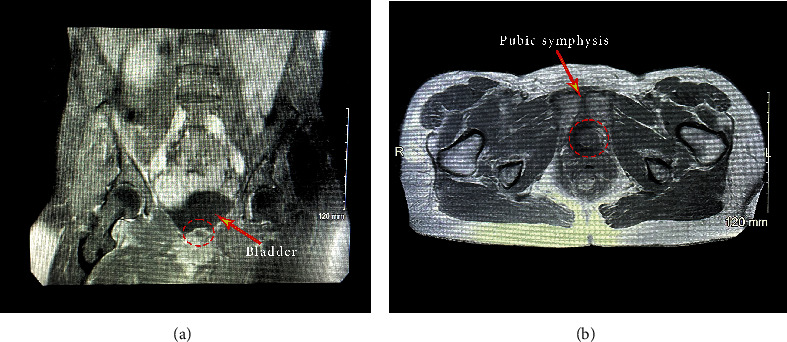
MRI of the pelvis demonstrating an undefined structure in the posterior inferior left paramedian bladder wall protruding into the bladder (red circle). Image demonstrates decreased T1 with minimal increased T2 with questionable minimal enhancement of a thin rim with no internal enhancement.

A 60-year-old Caucasian woman initially presented to the hospital emergency department complaining of a two-day history of right-sided inguinal pain and a fever of 103°F. The patient underwent workup in the emergency department. Laboratory workup was performed which showed a normal white blood cell count. Urinalysis was negative for pyuria or bacteriuria. Of note, the patient's C-reactive protein (CRP) was greater than 90 mg/L and erythrocyte sedimentation rate (ESR) was elevated at 47 mm/hr. A computed tomography (CT) scan of the abdomen and pelvis revealed inflammation of the iliopsoas muscle. Additionally, CT revealed an approximately 2 cm soft tissue density that appeared to lay dependently within or adjacent to a distended urinary bladder (Figure 1). An MRI of the pelvis was performed which revealed an undefined structure in the posterior inferior left paramedian bladder (Figure 2).

Following a diagnosis of sepsis secondary to a psoas abscess, the patient was admitted and treated with an intravenous course of vancomycin and piperacillin/tazobactam for four days. After completing her treatment course, the patients CRP and ESR had returned to baseline. The patient felt well, was hemodynamically stable, and was discharged 7 days after admission. She was instructed to follow-up with urology for further evaluation of the incidental bladder mass found on imaging.

**Figure 3:**
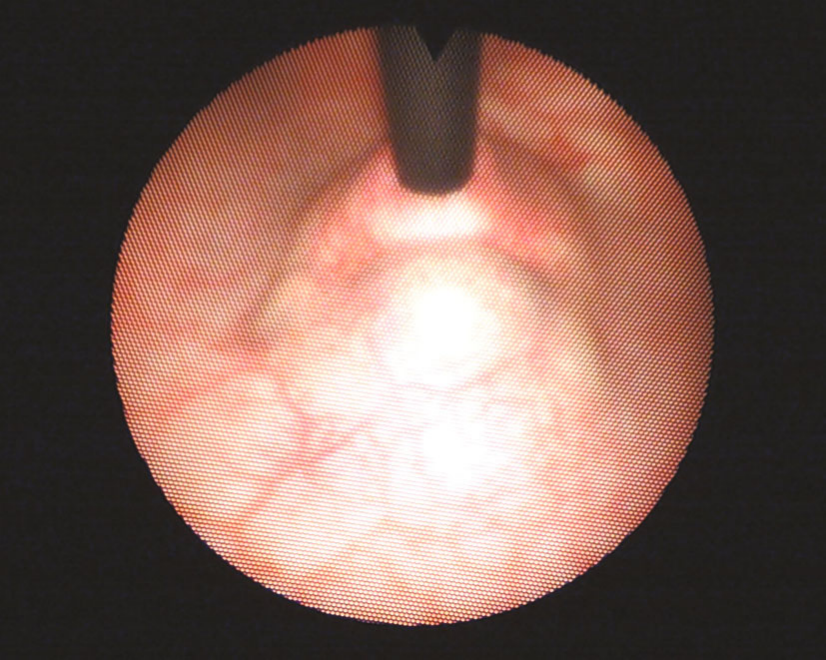
Cystoscopic photo of undefined, raised lesion in the bladder wall anterolateral to the urethra.

The patient presented to the urology clinic feeling well. Her past medical history included hypothyroidism and myocarditis secondary to COVID-19. The patient was nondiabetic and was not taking any immunosuppressive medications. She is a nonsmoker, and her family history was noncontributory. Her physical exam was unremarkable. As the initial part of the urologic workup, a cystoscopy was performed which demonstrated an undefined, raised lesion in the bladder wall anterolateral to the urethra at the bladder base (Figure 3). The mass did not exhibit the physical characteristics of a traditional neoplastic growth of the bladder. To exclude malignancy, the patient was then scheduled for a transurethral resection of the mass to obtain a specimen for pathologic evaluation.

**Figure 4:**
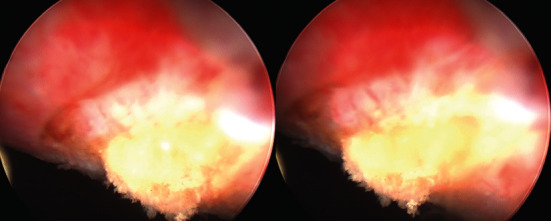
Photos taken during transurethral resection the bladder mass showing yellow, purulent fluid indicative of an abscess.

During transurethral resection of the mass, a yellow, purulent fluid was evacuated (Figure 4). The remainder of the bladder neck mass was shaved away in order to deroof the contents completely. The resectoscope was used to expel all residual pus until the bladder-wall cavity was cleared. A total of approximately 80 mL of purulent fluid was removed and sent for culture and cytology. Bladder tissue was sent for pathologic evaluation.

Following abscess deroofing and evacuation, a 16-French 2-way Foley catheter was placed. The patient was hemodynamically stable following the procedure and was discharged home where she completed a three-week course of trimethoprim/sulfamethoxazole. The patient was instructed to remove the Foley catheter at home, which she removed the day after the procedure.

Both wound and urine cultures demonstrated no growth. Cytology revealed mixed chronic and acute inflammatory cells in the background, mostly small lymphocytes with benign squamous and urothelial cells. Histologically, there was no significant smooth muscle tissue present. The surface urothelium showed focal small nests with bland cellular features, suggestive of mild proliferative chronic cystitis. Underlying connective tissues showed reactive fibrosis with some stromal hemorrhage. There was no evidence of papillary, in situ, or invasive malignancy.

Three weeks following deroofing of the bladder the abscess, the patient was reevaluated. She denied any lower urinary tract symptoms or suprapubic abdominal pain. Her physical exam was unremarkable.

## 3. Discussion

Bladder neck abscesses are an exceptionally rare finding. At the time of publication, the only case in the literature was reported in the Boston Medical and Surgical Journal in 1850 [1]. The patient described in this editorial was a young male who presented with significant dysuria, as well as a heavy, deep-seated pain in the perineum. Since then, there have only been reports of infected urachal cysts and intramural bladder-wall abscesses. Interestingly, bladder abscesses have a variable set of clinical features and presentations in healthcare settings as far as they have been reported.

In contrast to the aforementioned patient, our patient had no urologic history and was asymptomatic prior to being seen in the office. This difference could be explained by the anatomic differences between the female and male urinary tract. Obstructive lower urinary tract symptoms are much more common in men, and lesions that impact the area near the bladder neck are much more likely to cause symptoms [5]. In our patient, it is unclear how long she had the bladder lesion as she had no history of hospitalization or prior imaging. Her inguinal pain was most likely due to the iliopsoas abscess.

One possible explanation for the formation of multiple or recurrent abscesses is immunosuppression. It is well known that diabetic patients or those who are chronically immunocompromised from other conditions or medications are at a significantly increased risk for local and systemic infections [6]. However, our patient was nondiabetic and not on any immunosuppressive medications. Additionally, she had no history of organ transplantation or any condition that would have suppressed her immune system. Moreover, our patient had no history of urinary tract infections. This suggests that abscess formation in the urinary system may occur spontaneously in immunocompetent patients.

Due to the paucity of literature on bladder neck abscesses, it remains unclear what the disposition of our patient would have been without intervention. It is possible that following recovery from the psoas abscess, our patient could have remained asymptomatic for many years with the bladder abscess. Because her urine cultures were negative, it is unlikely that the bladder neck abscess would have led to hematogenous spread or sepsis without an obstruction. Our patient did well after the abscess evacuation indicating that surgical intervention may be a safe option in female with bladder neck abscesses.

## 4. Conclusion

Based on current evidence, the pathophysiological mechanism behind the formation of bladder abscesses is unclear. Drawing from the evidence of other abscesses, it may be appropriate to treat bladder abscesses with incision, drainage, and antibiotics. It remains unknown as to what the optimal treatment regimen is for bladder neck abscesses. Patients with iliopsoas abscesses with undefined lesions on imaging may warrant further investigation and workup. Incidental findings during cystoscopy may warrant further investigation in patients with comorbid abscesses.
